# Chronic Rhinosinusitis in Children

**DOI:** 10.1155/2012/573942

**Published:** 2011-10-05

**Authors:** Hassan H. Ramadan

**Affiliations:** Department of Otolaryngology, West Virginia University, P.O. Box 9200, Morgantown, WV 26506, USA

## Abstract

Rhinosinusitis is a very common disease worldwide and specifically in the US population. It is a common disease in children but may be underdiagnosed. Several reasons may account to the disease being missed in children. The symptoms in children are limited and can be very similar to the common cold or allergic symptoms. Cough and nasal discharge may be the only symptoms present in children. A high index of suspicion is necessary to make the diagnosis of rhinosinusitis in these children. The majority of those children are treated medically. Only a few number will require surgical intervention when medical treatment fails. Complications of rhinosinusitis, even though rare, can carry a high morbidity and mortality rate.

## 1. Introduction

Rhinosinusitis (RS) is a common disease in children that is sometimes overlooked. Children average 6–8 upper respiratory viral illness with 0.5–5% of these progressing to acute rhinosinusitis (ARS). An undefined number of these children will progress to have chronic rhinosinusitis (CRS) [1]. The disease has great impact on the health care system and the national economy as a whole [2]. 

The clinical symptoms of ARS in children include nasal stuffiness, colored nasal discharge, and cough with resultant sleep disturbance. Facial pain/headache can be present in older children. ARS is defined as symptoms lasting up to 4 weeks, subacute is when symptoms are between 4 weeks and 12 weeks, and CRS is when symptoms have been present for more than 12 weeks [3]. 

Rhinosinusitis is defined as a symptomatic inflammatory condition of mucosa of the nasal cavity and paranasal sinuses, the fluids within these sinuses, and/or the underlying bone [4]. The term “sinusitis” has been supplanted by “rhinosinusitis” due to evidence that the nasal mucosa is almost universally involved in the disease process [5].

## 2. Etiology and Pathogenesis

The etiology of CRS is a subject of much debate and ongoing research. The current hypothesis is that of a multifactorial pathogenesis. The paranasal sinuses are a group of paired, aerated cavities that drain into the nasal cavity via the sinus ostia. Several ostia drain in the middle meatus leading to the “osteomeatal complex” (OMC) as the focus of pathology [6]. Though the true anatomic role of the paranasal sinuses is uncertain, their ability to clear normal mucous secretions depends on three major factors: ostial patency, ciliary function, and mucous consistency [4, 7]. Any variety of inciting factors may irritate the sinus mucosa leading to inflammation, edema, bacterial proliferation, outflow obstruction, and mucociliary dysfunction. 

The association between CRS and allergic conditions, specifically rhinitis, has been well studied. Studies have shown that patients with persistent or seasonal allergic rhinitis had more significant radiographic findings of sinus disease [8, 9]. Further, CRS patients with concomitant allergic rhinitis have a significantly decreased rate of long-term success following surgical treatment [10]. 

Microbes have a controversial role in CRS. Though viral infections are known to precede episodes of viral rhinosinusitis [11], viral infections are not usually targeted as a part of CRS treatment. The use of antibacterial agents, however, has remained a first-line treatment for many practitioners despite the questionable role of bacteria. The paranasal sinuses, normally considered sterile, house a characteristic set of bacteria in CRS. A recent study showed that greater than half of CRS patients studied produced polymicrobial flora. The most common pathogens were those found in ARS. *Staphylococcus aureus* and coagulase-negative staphylococci were noted in those cultures from children with chronic disease. Anaerobes have been shown to be present in higher percentage in children with CRS [12]. The literature is replete with studies showing favorable patient response to treatment with antibiotics targeting these species, suggesting that there is some role for bacteria in CRS etiology [13]. 

The role of inflammatory mediators in the pathogenesis of CRS in children is still vague. In adults, emphasis is made on inflammatory response to the presence of bacteria rather than the action of microbes themselves. The finding of a sinus mucosal infiltrate of eosinophils, plasma cells, and lymphocytes suggests a process of “bacterial allergy.” In reality, there is likely a spectrum of illness ranging from an infectious etiology to a purely noninfectious inflammation [14].

Systemic factors can predispose to the development of CRS. Cystic fibrosis patients develop chronic mucosal inflammation and nasal polyps causing mechanical obstruction of sinus ostia [15]. Primary ciliary dyskinesia, though uncommon, is an example of CRS caused by a defect in a specific element of mucociliary clearance [16]. When clinically suspected, testing for the presence of allergy or any of the other above conditions will assist in tailoring a treatment regimen. 

## 3. Case Study

### 3.1. Chief Complaint

A 5-year-old girl presents to her primary care physician complaining of nasal stuffiness, colored nasal discharge, and cough for the past few weeks.

### 3.2. History

The patient has been stuffy and has been having a cough mainly at night that is keeping parents and child awake. This has been going on for some time over the past year. She does get treated with antibiotics; she seems to get better but then it starts again. Parents have been frustrated because of that. She was diagnosed with asthma and has been on inhalers and medications but with frequent exacerbations. She was tested for allergies, and she does not seem to have any, despite that she has been on nasal steroid sprays as well as oral antihistamines over the past several months. This current episode started a couple of weeks ago and has not resolved despite the oral antihistamines, nasal steroid sprays, and cold medicine. 

Her past medical history is negative. The child snores when sick, but no history of sore throats or ear infections. There is also no prior history of surgery. Grandparents do smoke but not near her.

### 3.3. Physical Examination

The patient is a healthy-appearing girl, very cooperative with normal vital signs. Heart and lung sounds are normal. Examination of the head and neck reveals a nasal septum deviated to the right. Anterior rhinoscopy shows bilateral hypertrophy of the inferior turbinates with mucopurulent discharge. Ears are clear with mobile tympanic membranes. Oral cavity is clear with no enlargement of the tonsils. No submucous cleft is noted.

## 4. Diagnosis

CRS in children is a clinical diagnosis. It is based on clinical presentation as well as duration of symptoms. In the first 7–10 days it is usually a viral one except if symptoms worsen and a complication develops. If symptoms persist and are not improving by 10 days, then acute rhinosinusitis needs to be entertained [17]. CRS is when symptoms persist beyond 12 weeks. Sometimes acute exacerbations of these symptoms can occur.Allergic rhinitis can present with a similar clinical picture and must be distinguished based on timing of symptoms, as well as presence or absence of purulence.

The physical examination of a patient suspected to have CRS involves direct visualization of the nasal cavity and associated structures. Anterior rhinoscopy with an otoscope is easy in children and should be a part of the initial evaluation. Attention is given to the nasal septum, all visible turbinates, and the presence of colored discharge. Nasal endoscopy in the older or more cooperative child may be very helpful.

Some authors have reported on the use of laboratory tests, including sedimentation rate, white blood cell counts, and C-reactive protein levels, to help diagnose acute sinusitis. These tests appear to add little to the predictive value of clinical findings in the diagnosis [18]. 

Imaging studies are not necessary when the probability of sinusitis is either high or low but may be useful when the diagnosis is in doubt, based upon a thorough history and physical examination. Plain sinus radiographs may demonstrate mucosal thickening, air-fluid levels, and sinus opacification. A CT scan is necessary when a complication is suspected, in children with polyps, or in those children who failed medical therapy and are considered for surgery [1].

## 5. Treatment

The case presented meets the diagnostic criteria for CRS based on her complaints of nasal stuffiness, colored nasal discharge, and cough with acute exacerbations for greater than 12 weeks, as well as a finding of colored discharge on anterior rhinoscopy.

The goal of treatment in rhinosinusitis is to reduce the mucosal inflammation thus relieving the blockage of the ostia and impairment of mucociliary flow that is the hallmark of the disease. 

Empiric treatment with a course of orally administered antibiotics has been a mainstay of treatment. For ARS a 10–14 days course of oral amoxicillin is first line of treatment. If the patient dose not get better in 48–72 hours, then antibiotic should be changed to amoxicillin with clavulanic acid. Three to four weeks of amoxicillin with clavulanic acid is a first-line choice for CRS or those children with acute exacerbation of CRS because of adequate penetration of the sinus mucosa and efficacy against *S. aureus* and anaerobes [19]. Antibiotic choice is largely guided by patient tolerance. However, in adult cases of treatment failure, culture and sensitivity from sinus secretions can guide antibiotic choice [20]. Since this cannot be performed in children in the office, performing those cultures in the operating room can be an option. Obtaining cultures is not the standard of care for initial therapy, however. There is a lack of evidence comparing randomized head-to-head efficacy of the various antibiotic classes or demonstrating a benefit of multiantibiotic regimens. 

Initial medical management also includes a regimen of topically applied corticosteroids. Fluticasone, beclomethasone, budesonide, and mometasone are popular choices. Topical corticosteroids have been shown to downregulate the inflammatory cytokine profile of sinus mucosa and improve subjective patient symptoms [21–23]. Though uncommon, patients should be aware of local side effects of mucosal drying and bleeding. Often, the choice of agent simply depends on local practice patterns. Duration of therapy is up to 3 months, and patient response is unlikely before 2 weeks of use. Systemic absorption of topical agents is minimal, but there is evidence that using metered-dose inhalers, rather than spray bottles, prevents accidental overdose and subsequent adrenal suppression [21]. Systemic steroids in burst or taper are generally avoided due to side effects. They do have a role in patients with significant polyposis, as this may physically prevent the delivery of topical agents to the site of action. Saline nasal irrigation with a 2-3% solution has been found to be helpful. Daily irrigation has been shown to significantly decrease symptoms and improve QOL scores [24, 25]. Relief is likely due to improved mucous outflow and a decrease in secretions and load of inflammatory mediators. 

Some clinicians prescribe the use of nasal decongestants for symptomatic use. These agents, however, should not be used for longer than 3-4 days because they are relatively short acting and can cause rebound congestion with chronic use. There is also ongoing research into the use of antileukotrienes. Although they are theoretically effective in situations of eosinophilic inflammation, substantial evidence is lacking [16]. Future directives in medical management include the use of immunotherapy to decrease inflammation, especially in patients with recalcitrant disease and those with concurrent allergic disease. 

## 6. Treatment Failures

In cases of treatment failure, a referral to an otolaryngologist can be helpful for further treatment and possibly surgical intervention. Other indications for referral include the presence of severe signs including bleeding, orbital symptoms, facial swelling, uncertainty regarding diagnosis, or the presence of nasal polyposis. 

**Figure 2:**
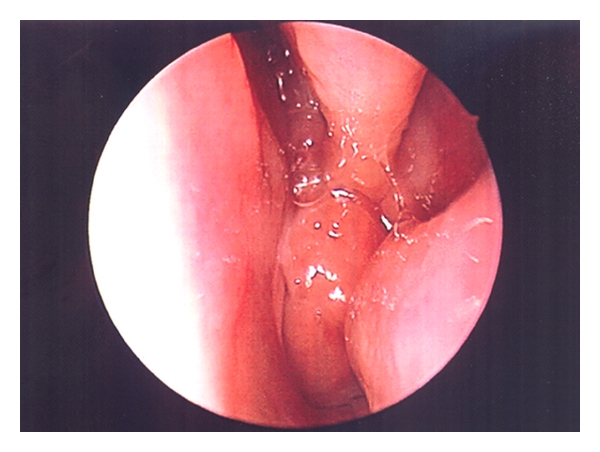
An endoscopic view using a 2.7 mm zero-degree rigid scope showing enlarged adenoids.

Nasal endoscopy in a cooperative child and the older child in the office can be very informative. Anatomical abnormalities, nasal polyps, and adenoid hypertrophy are findings that will help with further management of the child (Figure 2). 

Based on the exam findings, a CT scan can be obtained. It is important that the scan is obtained after at least 3-week course of antibiotics. Plain films of the sinuses are not helpful because of low sensitivity and specificity. MRI does have a place in the evaluation of complications, soft tissue involvement, and suspected neoplasia.

**Figure 3:**
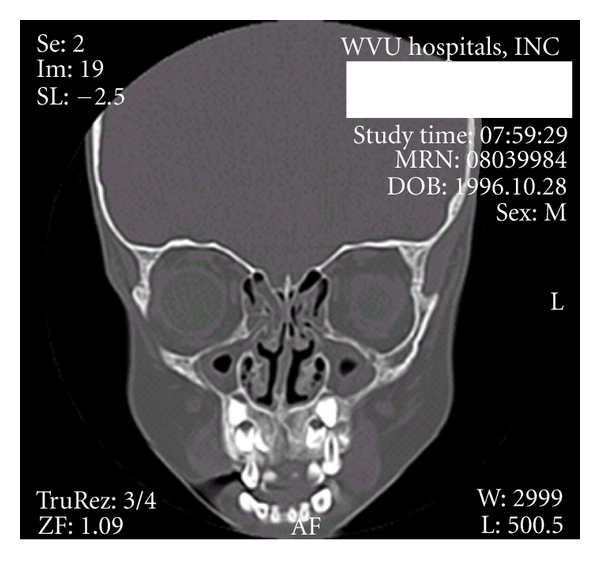
Coronal CT scan of the sinuses demonstrating significant disease in both ethmoids and maxillary sinuses with blockage of the OMC.

After referral for further evaluation, our patient had mucopurulence bilaterally. After treatment with 20 days of antibiotics and topical nasal steroids with nasal saline washes, a CT scan was obtained, and it showed blockage of her osteomeatal complex area with mucosal thickening in both maxillary sinuses (Figure 3). 

## 7. Surgical Management

Surgical management for CRS that failed medical therapy in adults is ESS [26]. In children, however, we have several surgical options to consider prior to ESS. Most advocate an adenoidectomy as an initial step in the surgical management of those children. The success rate however is in the 50–60% [27, 28] range which is less than the 87% success rate with ESS [29]. Interestingly, a recent study comparing biofilm presence of adenoid samples from children with CRS found an incidence of 95% biofilm present in those samples compared to only 2% in adenoid samples of children with sleep apnea. This may partially explain the importance of removing the adenoids in those children with CRS [30]. Otolaryngologists are reluctant to proceed with ESS as a 1st line of treatment because of the fear of facial growth retardation [31]. Despite the fact that one study showed no difference in facial growth 10 years after ESS in children compared to a comparative group of children who had no surgery, that concern is still there [32]. Because of the average success rate with adenoidectomy alone, a sinus wash at the time of adenoidectomy has been advocated. The procedure will flush the sinuses, and at the same time a culture for antibiotic guidance would be obtained. Success rate with this procedure was 88% [33]. 

After a detailed review of those children who failed adenoidectomy was reviewed, it was noted that children with asthma or severe sinus disease as noted by their CT scan score had the worst outcome. Based on that information, subsequent studies have noted that children with asthma and a more severe disease had a much improved outcome if a sinus wash or sinus surgery was performed at the time of adenoidectomy [34]. 

Functional endoscopic sinus surgery (FESS) is a term for minimally invasive procedures designed to restore the natural drainage pathways of the paranasal sinuses [26]. FESS is performed under general anesthesia, typically as a same-day procedure. The nasal cavity is directly visualized, and various specialized tools are used to relieve obstructive lesions of sinus outflow including polyps and diseased mucosa. The affected sinus air cells are opened in a manner that augments natural mucociliary outflow. The procedure is usually a conservative one in those children [35]. 

FESS is indicated in patients with CRS who fail medical therapy or demonstrate complications of disease. Surgical complications are rare and are usually related to damage to adjacent structures including the orbital contents and the skull base. Regular postoperative followup is essential. Medical management with antibiotics and intranasal steroids may continue postoperatively, specifically if the children have allergic rhinitis.

## 8. Outcomes and Summary

Our patient had an adenoidectomy with a sinus wash of her maxillary sinuses, since she had severe asthma and severe disease based on her CT scan. There were no operative complications. Postoperatively she was given culture-directed oral antibiotics for 14 days. At-six-month followup, there was no reported sinus infections and her symptoms were much improved. She continues to use her topical nasal steroids. Her asthma was much improved that her parents reported discontinuation of all of her asthma medications.

## 9. Conclusions

CRS is a common disease in children which was shown to have significant impact on the quality of life of these children. In the majority of cases medical treatment is very successful; however, in a small percentage surgical treatment may need to be entertained specifically in those children with asthma. Proper patient selection, counseling, and followup are essential for a favorable surgical outcome.

## Figures and Tables

**Figure 1:**
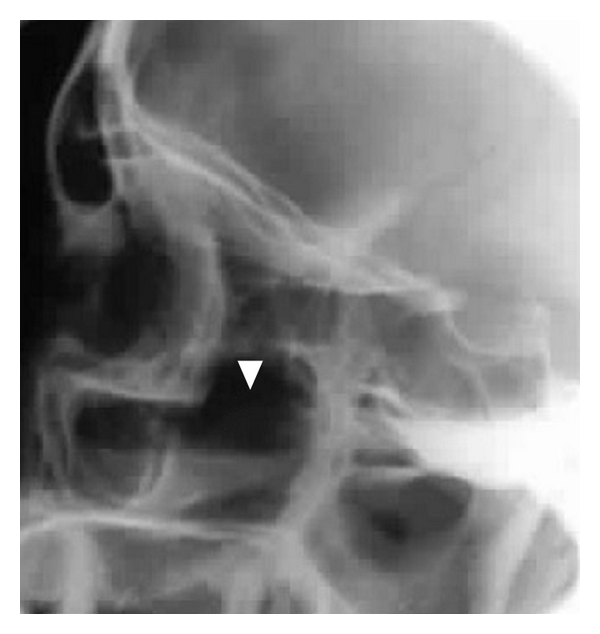
Lateral plain X-ray showing air-fluid level in the maxillary sinus.
